# Rotavirus in Calves and Its Zoonotic Importance

**DOI:** 10.1155/2021/6639701

**Published:** 2021-04-21

**Authors:** Umer Seid Geletu, Munera Ahmednur Usmael, Fufa Dawo Bari

**Affiliations:** ^1^College of Agriculture, Animal Science Department, Oda Bultum University, P.O. Box 226, Chiro, Ethiopia; ^2^West Hararghe Zone, Chiro Wereda, Animal Health Worker, P.O. Box 226, Chiro, Ethiopia; ^3^Department of Microbiology, Immunology and Veterinary Public Health, College of Veterinary Medicine and Agriculture, Addis Ababa University, P.O. Box 34, Bishoftu, Ethiopia

## Abstract

Rotavirus is a major pathogen responsible for diarrheal disease in calves, resulting in loss of productivity and economy of farmers. However, various facets of diarrheal disease caused by rotavirus in calves in the world are inadequately understood, considering that diarrheal disease caused by rotavirus is a vital health problem in calves that interrupts production benefits with reduced weight gain and increased mortality, and its potential for zoonotic spread. The pathological changes made by rotavirus are almost exclusively limited to the small intestine that leads to diarrhea. It is environmentally distributed worldwide and was extensively studied. Reassortment is one of the important mechanisms for generating genetic diversity of rotaviruses and eventually for viral evolution. So, the primary strategy is to reduce the burden of rotavirus infections by practicing early colostrum's feeding in newborn calves, using vaccine, and improving livestock management. Rotaviruses have a wide host range, infecting many animal species as well as humans. As it was found that certain animal rotavirus strains had antigenic similarities to some human strains, this may be an indication for an animal to play a role as a source of rotavirus infection in humans. Groups A to C have been shown to infect both humans and animals. The most commonly detected strains in both human and animals are G2, G3, G4, and G9, P [6]. Therefore, this review was made to get overview epidemiology status and zoonotic importance of bovine rotavirus.

## 1. Introduction

Bovine rotavirus is the most recognized pathogen causing acute diarrhea in calves under one month of age worldwide [1, 2]. It has also been recognized as the major pathogen of acute diarrhea in both humans and animals. So, it has the potential of zoonotic and economic impact [3]. Infection appears and spreads rapidly, causing extensive damage to the intestinal lining which results in rapid fluid loss and dehydration [4]. Genetic reassortment is one of the important mechanisms for generating genetic diversity of rotaviruses and eventually for viral evolution. There is no treatment for BRV, but early and confirmatory diagnosis helps to make appropriate prevention and control measures, which could prevent the great economic losses to farmers and the livestock industry [5].

Eight percent of diarrheic calves tested were positive for at least one of the target enteric pathogens (like bovine coronavirus, bovine rotavirus, bovine norovirus, and bovine torovirus), suggesting that the infectious factor is still a major cause of calf diarrhea [6]. The majority of diarrheic cases were identified among 0 to 4-week-old calves. A successful dairy and beef farm operation requires that a large percentage of cows wean a live healthy calf every year. Rearing healthy dairy calves to weaning time requires maximizing the calf's level of immunity against disease, while minimizing its exposure to infectious agent. However, among the factors that have been hindering success of dairy and beef industry, morbidity and mortality of calves are the ones that cause major concern. Morbidity and mortality are important causes of economic losses on dairy farms worldwide. In spite of advancement made in dairy and beef husbandry practices, clinical medicine, and diagnostic techniques, the morbidity and mortality rates of dairy and beef calves are still unacceptably high even on many advanced dairy farms in developed countries [7]. Thus, it is necessary to identify risk factors that are responsible for dairy and beef calf morbidity and mortality in order to design and implement preventive measures.

Rotavirus is one of highly distributed disease agents worldwide and has been extensively studied [8]. In different studies, BRV infection rates of 20–60% in samples of diarrhea have been reported [9]. Prevalence of rotavirus ranges from 11.8% to 26.8% in India among diarrheic calves [10]. Also, in European countries rotavirus infection was widely examined. In Sweden between 1993 and 2006, estimated prevalence was 24–47% [11], 42% in diarrheal outbreaks in the UK [12], and 37 to 47.4% in France [13]. In Asian countries like Bangladesh, prevalence of rotavirus infection in calf feces varied from 0 to 7% [14]. In Ethiopia, the prevalence of rotavirus was 16.7% [15].

To know the epidemiology status, zoonotic importance, and other related information about rotavirus in calves is very important to develop different strategies for control and prevention of rotavirus infection of calves and humans. This review provides an overview of epidemiological status and zoonotic importance of bovine rotavirus. This is needed for planning a proper control and preventive measure in the country.

## 2. Rotavirus: Overview

Rotavirus was initially reported in 1972 in Australia [16]. The virus was recognized by direct electron microscopy visualization in the duodenal biopsies of a child with acute diarrhea and named duovirus. The virus was named rotavirus because of its characteristic wheel-shaped (rota is a Latin word which means wheel) morphology when seen under an electron microscope [17].

### 2.1. Virology of Rotavirus

#### 2.1.1. Structure and Its Genome

Bovine rotavirus (BRVs) is a primary etiological agent of calf diarrhea. Rotaviruses are double-stranded RNA (dsRNA) held in the inner core of the three-layered virus. Rotavirus is a nonenveloped virion possessing 11 dsRNA segments which a size range 16∼21 kilo base pairs within the family Reoviridae and is very stable over a wide pH range with heat liability. There are seven serogroups (A-G) of rotaviruses based on antigenic and genetic similarities of the intermediate capsid protein of VP6. Group A rotaviruses are the major cause of rotavirus infection in domestic animals and, initially known as neonatal calf diarrhea virus, were one of the first identified viral causes of diarrhea [4]. Most BRVs (95%) belong to group A, although groups B and C rotaviruses have also been identified in field cases [18].

Genome segments code for structural proteins found in the virus particle and the nonstructural proteins found in infected cells but not part of the mature particles. The genome consists of 18,555 nucleotides in total. Each segment is a gene, numbered 1 to 11 by decreasing size. The segmented genome can be separated by polyacrylamide gel electrophoresis (PAGE) to reveal an RNA migration pattern or electropherotype. The RNA pattern is both constant and characteristic for a particular strain and has been widely used in epidemiological studies for monitoring the transmission and spread of rotavirus [19].

#### 2.1.2. Proteins

**Figure 1:**
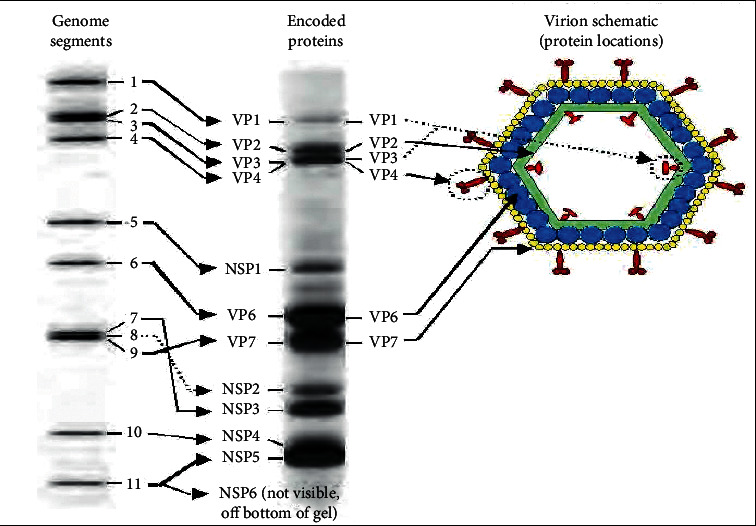
Diagrammatic representation of the rotavirus particle and its genome coding. (I) The migration pattern of 11 dsRNA genome segments of rotavirus on a polyacrylamide gel. (II) Virus proteins encoded by specific genome segments in Section 1. The proteins were blotted onto a cellulose membrane and detected with rotavirus-specific antibodies. (III) Schematic diagram of rotavirus particle showing the cross section arrangement of viral proteins through the three capsid layers namely: outer (VP4, red; VP7, yellow), inner (VP6, blue) and the inner core (VP2, green). Source: [23].

The nomenclature of the viral proteins designates the structural proteins as VP and nonstructural proteins as NSP followed by sequential numbering from 1 to 6 [20]. Analysis of gene encoding segments shows that there are six structural proteins (VP1 to VP4, VP6, and VP7) and six nonstructural proteins (NSP1 to NSP6). The structural proteins build up the viral particle (Figure 1) and the NSPs have function in either the viral replication cycle or interaction with host proteins to influence the pathogenesis or immune response. Each of the 11 segments of dsRNA encodes a single viral protein except segment 11 which encodes two proteins [21]. Figure 1 summarizes the six structural (VP) and six nonstructural protein (NSP). The functions of each protein are summarized in Figure 1 and Table 1.

The proteins encoded by the rotavirus genes are well established. Except for segment 11, which encodes for two proteins NSP5 and NSP6, the rest of the segments encode a single protein. The six viral proteins (VP1, 2, 3, 4, 6, and 7) form the virus particle (virion). VP1 is the RNA-dependent, RNA polymerase for rotavirus, located in the core of the virus particle [24]. VP2 is a replication intermediate, forms the core layer of the virion, and binds the RNA genome while VP3 is an enzyme guanylyl transferase that catalyses the formation of the 5′ cap in the posttranscriptional modification of mRNA. VP4 determines the rotavirus P serotype as well as host specificity, virulence, and protective immunity; it also binds to molecules on the surface of cells called receptors and drives the entry of the virus into the cell [25]. VP6 is highly antigenic and can be used to identify rotavirus species and it also determines the A-G groupings, and I, II subgroupings of rotavirus. VP7 is a glycoprotein that determines the G serotype and that is involved in immunity to infection [26].

The six nonstructural proteins (NSP1, 2, 3, 4, 5, and 6) are only produced in cells infected by rotavirus [21]. NSP1 binds interferon regulatory factor 3 and may inhibit interferon response during rotavirus infection. In conjunction with NSP5, NSP2 is involved in the synthesis and packaging of viral RNA and creation of viroplasms and is required for genome replication. NSP3 binds viral mRNA at the 3′ end, promotes viral protein synthesis, and is responsible for the shutdown of host cell protein synthesis. NSP4 is a viral enterotoxin and induces diarrhea during infection. NSP6 is an RNA binding protein encoded by gene 11 from an out-of-phase open reading frame [27].

In comparison to most cellular mRNAs, rotavirus mRNAs are unique in that they contain 5′-terminal caps but lack 3′-terminal poly (A) tails. During replication, the viral mRNAs serve two functions: (i) direct synthesis and (ii) acting as templates for the synthesis of minus-strand RNAs to produce dsRNAs [28]. The synthesis of dsRNAs is an event that follows or occurs simultaneously with the packaging of mRNA templates, as naked dsRNA cannot be detected in infected cells. Likewise, the absence of free dsRNA in the infected cell indicates that dsRNA remains particle associated once synthesized. Given that the 11 genomic dsRNAs are present in equimolar concentration in both infected cells and virions, the packaging and replication of the 11 species of viral mRNAs into dsRNAs must be a highly coordinated process [29].

Both outer capsid proteins VP7 and VP4 (the spike protein) are targets for neutralizing antibodies. VP4, VP6, and VP7 play a major role in maintaining viral structure, virus attachment, and antigenicity. Although early studies implicated VP7 in the cell entry process, subsequent studies increasingly have indicated that VP4 is the major player in this process. VP4 is susceptible to proteolysis and viral infectivity is increased several folds when VP4 is proteolytically cleaved and facilitates virus entry into cells [22]. During proteolysis, VP4 is cleaved into VP8 ^*∗*^ (amino acids 1 to 247) and VP5 ^*∗*^ (amino acids 248 to 776), and the cleavage products remain associated with the virion [30].

#### 2.1.3. Classification and Serogroups

Based on the group specific epitopes localized in an immunodominant site of VP6 between amino acid residue 48 and 75, rotaviruses have been divided into five serological species (A-E) and two additional tentative species (F and G) according to the International Committee on Taxonomy of Viruses (ICTV) [31]. These rotavirus species are commonly referred to as rotavirus groups. Rotaviruses belonging to groups A, B, C, and H (RVA, RVB, RVC, and RVH, respectively) have been associated with acute gastroenteritis in humans and animals, whereas groups D, E, F, and G (RVD, RVE, RVF, and RVG, respectively) rotaviruses are known to infect only animals, mostly birds [32]. A novel tentative group I was recently described in Hungarian sheltered dogs [33].  Table 2 is summarized the rotavirus group with respective host species.

Group A rotaviruses (RVA) can be further classified into P or G types based on genetic and antigenic similarities of VP4 and VP7. VP4 (P protein for “protease-sensitive” due to its trypsin mediated cleavage required for virus adsorption into cells) determines the P serotypes. VP7 (G protein for “glycoprotein” forming the matrix of the capsid) defines G serotypes [26]. For G types, serotypes (determined by neutralization assay) and genotypes (determined by RT-PCR) are largely identical, thereby allowing the use of the same numbering system. For P types, more genotypes than serotypes have been identified, owing to lack of monospecific P antisera. As a result, P types are identified as serotypes by Arabic numbers and as genotypes by Arabic numbers in square brackets. Thus, the serotype of prototype human rotavirus strain Wa is described as G1P [8]. To date, at least 27 G types and 37 P types have been found in humans and animals [31, 35]. Unlike P types, correlation between G serotypes and genotypes is complete. Therefore, where available, P serotypes and genotypes are designated jointly with genotypes in square brackets, for instance, RVA/Human-tc/USA/DS-1/1976/G2P1B [4] [31].

Although the dual typing system has been widely used in most epidemiological and molecular characterization studies, its use is primarily limited to classifying rotavirus strains. The dual typing system cannot determine factors that are involved in viral tropism and virulence of rotavirus strains. Furthermore, some evolutionary pathways like reassortment and recombination followed by all the 11 genome segments of rotaviruses cannot be studied because the dual classification is restricted only to outer capsid encoding genome segments [36].

In addition to the G and P genotyping of rotavirus, a whole genome-based genotyping system was recently proposed based on the assignment of genotypes to all the 11 gene segments (i.e., G/P and non-G/P genes) [36]. In the new genotyping system, the acronym Gx-P [x]-Ix-Rx-CxMx-Ax-Nx-Tx-Ex-Hx, where x is an integer, defines the genotype of the VP7-VP4-VP6-VP1-VP2-VP3-NSP1-NSP2-NSP3-NSP4-NSP5 genes of a given rotavirus strain. Following the advent of hybridization techniques, researchers could investigate the occurrence of reassortment events between human strains that belong to different genogroups or between human and animal strains which frequently lead to generation of novel rotavirus strains. Human rotaviruses were classified into two major (represented by the Wa and DS-1 reference strains) genogroups and one minor (represented by the AU-1 reference strain) genogroup [37].

The Wa-like strains are characterized by non-G/P genotypes (I1-R1-C1-M1-A1-N1-T1- E1-H1) and tend to have G/P genotypes G1P [8], G3P [8], G4P [8], or G9P [8] [38]. In contrast, the DS-1-like strains are characterized by non-G/P genotypes (I2-R2-C2-M2- A2-N2-T2-E2-H2) and tend to have G/P genotype G2P [4]. The third minor AU-1-like strains are characterized by non-G/P genotypes (I3-R3-C3-M3-A3-N3-T3E3-H3) and tend to have G/P genotype G3P [9]. Whole genome-based analysis is a reliable method for obtaining conclusive data on the origin of an RVA strain and for tracing its evolutionary pattern [36]. RVA of VP7 and VP4 genotypes with their respective host species are summarized in Table 3.

Rotavirus surveillance also generates valuable data on the circulating rotavirus strains (Table 4). These data are vital to improving vaccine development tracking emergent types, and helping to assess vaccine effectiveness, and changes in strain diversity after vaccines are introduced. Globally, G1, G2, G3, G4, and G9 are the most prevalent VP7 serotypes; P [4], P [6], and P [8] are the most common VP4 genotypes, and G1P [8], G2P [4], G3P [8], G4P [8], and G9P [8] comprise 70–90% of circulating rotavirus strains [39, 41]. In Taiwan, G1 (40%), G3 (27%), G9 (18%), and G2 (8%) are the most common VP7 serotypes [42]. G6 and G10 type are reported to be the most prevalent in cattle [43]. The geographic distribution of rotavirus serotypes is summarized in Table 4.

#### 2.1.4. Reassortment and Antigenic Variation

Reassortment is one of the important mechanisms for generating genetic diversity of rotaviruses and eventually for viral evolution. Although host species barriers and host range restriction exist in rotavirus, reassortment can result in interspecies transmission, which also contributes to the diversity and evolution of rotavirus. A crucial factor in the generation of reassortant viruses is the frequency of coinfection. In developing countries, the rate of RV coinfection can be as high as 20%, while in developed countries, the rate is typically less than 5% [44]. It may be because of the high rate of coinfection that the genetic diversity of viruses in developing countries can be so much higher than in developed countries [44]. Due to the high frequency of coinfection, large genetically distinct RV clades may not be detectable in some developing countries [44].

Sequence analysis has shown that the antigenic epitopes of VP7 and VP4 proteins assigned to the same G and P type, respectively, will frequently show amino acid variation [45]. This has been seen for VP7 and VP4 proteins of viruses recovered from different countries in the same year or that belong to different cocirculating clades at one site. Such amino acid variation may ultimately have an impact on vaccine efficacy, particularly if protection is based chiefly on G and P type specific homotypic responses. The effective titer of a G type specific neutralizing antiserum is affected by the amino acid composition of VP7 antigenic epitopes, even if the VP7 proteins are of the same G type [46].

#### 2.1.5. Replication

Viruses interact with the host at all stages of replication: cell entry, viral transcription, translation, genome synthesis and packaging, and cell exit. These interactions not only are important for producing new virus progeny, but also enable the host to recognize the presence of an infectious agent. As host species have evolved mechanisms to defend against pathogens, viruses have in turn evolved strategies to avoid the host immune response [47].

**Figure 2:**
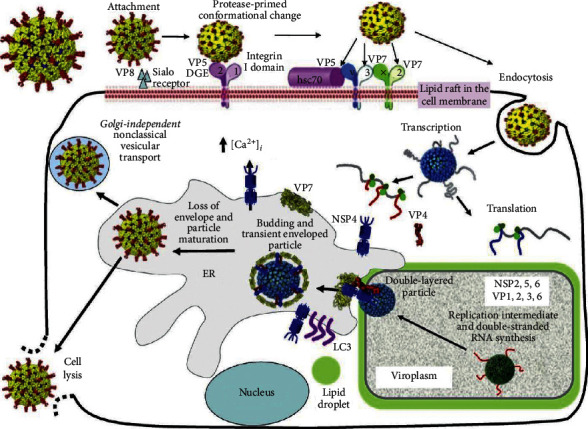
The rotavirus replication cycle. Source: [32].

Rotavirus replication takes place in the cytoplasm of infected cells, in viroplasms, electron dense structures near the nucleus and ER [48]. Newly made viruses budded out from viroplasms into ER, through binding to the tail of the ER transmembrane viral glycoprotein NSP4. Although the virus replication process includes synthesis and transport of glycoproteins, the Golgi apparatus is not involved in rotavirus replication. Instead, rotavirus replication, morphogenesis, and pathogenesis are regulated by intracellular calcium concentrations. The rotavirus toxin NSP4 has been shown to be released very early during an infection, first as a cleavage product including the toxic region released from infected cells, starting at 4 hours after infection and later during infection as fully glycosylated NSP4. Based on cell culture studies, the general steps of rotavirus replication are as follows [48] (Figure 2).

Virus attaches to the cell surface by VP4 or the cleavage product VP8. The conformational change is protease-dependent, where VP4 is cleaved into VP8 and VP5. Rotavirus has tropism for mature enterocytes but the exact receptor for viral binding in vivo has not yet been identified, although sialic acid, integrins, histo-blood group antigens [49, 50], and toll-like receptors (TLR) have been suggested. Cell entry, by receptor-mediated endocytosis occurs via VP5, thus indicating that cleavage of VP4 into VP5 and VP8 is required. Calcium dependent endocytosis has also been shown. Nonclathrin, noncaveolin-dependent endocytosis delivers the virion to the early endosome. It has also been suggested that rotavirus can enter the cell by direct entry or fusion. Uncoating of the TLP, reduced calcium concentrations in the endosome are thought to trigger the uncoating of VP7 and loss of the outer capsid (VP7, VP5, and VP8). Double-layered particles (DLP) (core proteins and inner capsid VP6) are released into the cytosol [51].

Transcription and translation take place in the cytoplasm of the cell. The internal polymerase complex (PC) (VP1 and VP3) starts to transcribe capped (+) RNAs from each of the eleven dsRNA segments. (+) RNA serves either as mRNA for direct translation, synthesis of viral proteins by cellular ribosomes, or as a template for (−) RNA synthesis of viral genome replication, taking place in viroplasm. Assembly is the NSP2 and NSP5 interact to form viroplasms, where replication and sub-viral particle assembly takes place. DLPs are formed within the viroplasms. The assembly process of the outer capsid is not fully understood but it is thought that the transmembrane protein NSP4 recruits DLPs and the outer capsid protein VP4 to the cytosolic side of the ER membrane. The NSP4/VP4/DLP-complex then buds into ER. The removal of the ER membrane and NSP4 takes place in the ER through interaction with ER-resident VP7 and the final TLP is formed. Virus release from the infected cell is through cell lysis or Golgi-independent nonclassical vesicular transport. In the GIT, the virion will be exposed to trypsin-like proteases, which will cleave the protease-sensitive VP4 into VP5 and VP8, thus resulting in a fully infectious virion [48].

### 2.2. Epidemiology of Rotavirus and Geographical Distribution

#### 2.2.1. Epidemiology of Rotavirus in Animals

Rotavirus can cause a diarrhea and lead is a serious welfare problem in calves and a cause of economic loss due to mortality, treatment costs, and poor growth. Rotavirus is highly infectious because (1) virus particles are present in very large numbers (10^10^–10^12^ particles/ml) in infected feces and (2) the virus is resistant to inactivation and can remain infectious for 9 months at room temperature or for 1 hr at 60°C. Furthermore, rotaviruses are not easily inactivated by the commonly used disinfectants. Rotavirus surviving in a contaminated environment from one calving season to the next may therefore be the source of infection in an outbreak. However, adults are the major source of infection for calves. Whatever the source of the virus, infection spreads predominantly by fecal-oral contact [52]. Calves most often become infected with rotavirus during the first week of life.  Table 5 summarizes some study of rotavirus in animals in different parts of the world.

#### 2.2.2. The Status of Rotavirus in Human and Animals in Ethiopia

Ethiopia is one of the five countries with the greatest human rotavirus burden worldwide and accounts for 6% of all rotavirus deaths globally [63]. It is estimated that 28 percent of all under-five diarrheal disease hospitalizations in Ethiopia are caused by rotavirus [64]. Also some study showed, among children <5 years of age, rotavirus prevalence ranging within 18%–28% of diarrhea hospitalizations [65]. In a cross-sectional study carried out in Jima Hospital, Ethiopia, to reveal the prevalence of rotavirus infection among 154 infants and young children, rotavirus was detected in 26.6 % of fecal specimens and 90.2% (37/41) occurred in children under 2 years. The highest rate of rotavirus antigen detection was observed among the 7–12 months age group (34%) [66].

In a study to see the epidemiology of rotavirus and norovirus in Awassa, southern Ethiopia from 200 under-five children with diarrhea 2008-2009, the prevalence of rotavirus was 22% and the genotyping showed G3P [6] (48%, globally uncommon strain), G1P [8] (27%), and G2P [4] (7%) being the strains most commonly identified. Data from hospital-based surveillance of rotavirus gastroenteritis among children less than five years from 2007 to 2011 in Addis Ababa, Ethiopia, showed that rotavirus was prevalent in 20% of children enrolled from 1,749 diarrheal samples collected in the five-year period. Another study showed the prevalence of rotavirus 25% in children less than five years in northwest Ethiopia by Gelaw et al. [67]. Only two reports were found in Ethiopia by Abraham et al. [15] and Geletu et al. [68] that indicated presence of 16.7% and 7.2% in calves in central Ethiopia, respectively.

### 2.3. General Pathophysiology

The severity and localization of rotavirus infection vary among animal species and between studies, but pathological changes are almost exclusively limited to the small intestine. Rotavirus infects the mature nondividing enterocytes in the middle and top parts of the villi in the small intestine [69]. At the cellular level, the infection is characterized by vacuolization, blunting, and shortening of the villi. Rotavirus also produces the enterotoxin NSP4, which is thought to play an important role in the pathophysiology and clinical symptom of rotavirus disease [70–72]. The incubation time is 24 to 48 hours and illness usually lasts from 3 to 5 days, longer in immune-compromised individuals [73]. There are few pathology studies of the duodenal mucosa of infants infected with rotavirus. Biopsies have displayed shortening and atrophy of villi, distended endoplasmic reticulum, mononuclear cell infiltration, mitochondrial swelling, and loss of microvilli [74]. Systemic spread of rotavirus has been reported but is very rare and its clinical importance remains unclear. In a few cases, rotavirus RNA has been detected in cerebrospinal fluid (CSF) [75], possibly associated with meningitis, encephalopathy, and encephalitis [76].

### 2.4. Pathogenesis of Rotavirus Infection

Bovine rotaviruses group A are enteropathogenic agents more commonly associated with neonatal diarrhea in calves up to 30 days old [1]. The mechanism of rotavirus-induce diarrhea is not completely known. The major mechanism appears to be a decreased absorption of salt and water related to selective infection of the absorptive intestinal villous cells, resulting in net fluid secretion. The main place for rotavirus infection is brush border of villous epithelial cells in the small intestine. The infected cells are rapidly replaced with undifferentiated crypt cells, and this results in reducing activity of lactase in villous [77].

The primary mode of transmission of rotavirus is fecal-oral, although some studies have reported low titers of virus in respiratory tract secretions and other body fluids, indicating the possibilities for airborne and waterborne transmissions of rotavirus [78]. After ingestion, the rotavirus particles exclusively infect the mature differentiated enterocytes in the middle and upper part of the villi of the small intestine leading to structural changes in the intestinal epithelium [69]. The virus replicates in the cytoplasm of epithelial cells of the mature absorptive and enzyme producing enterocytes of small intestinal villi. Destruction of mature entrecotes in the villi leads to rupture and sloughing of the enterocytes with release of virus to infect adjacent cells. Unlike the parvovirus, rotavirus can infect neither the immature villous crypt cells nor the colonic enterocytes. Rotavirus attaches to its cellular receptors (sialoglycoprotein and integrins) via the VP4 protein. The virus is thought to invade target cells in two possible ways, by direct entry or fusion with enterocytes and through Ca^2+^-dependent endocytosis [79].

Rotavirus may cause diarrhoea by three different mechanisms. First, within 12–24 hours after infection, enterocytes are intact but the levels of the brush-border disaccharidases (sucrase, maltase, and lactase) are greatly reduced. As a result, disaccharides in the diet cannot be hydrolysed to monosaccharides and thus cannot be absorbed, leading to osmotic diarrhoea [21]. Second, NSP4 has an effect in opening calcium channels in the enterocytes. This causes an efflux of sodium and water, producing secretory diarrhea [80]. Finally, the higher intraenterocyte calcium concentration causes enterocytes to die by oncosis. The rate of death of the mature villous tip enterocytes exceeds the rate of growth of immature enterocytes that are regenerated from the stem cells in the crypt, causing villous blunting and thus malabsorption [22]. Infection resolves both as the virus runs out of susceptible mature enterocytes and an immune response is generated [69].

The sign of emesis, which is a hallmark of the rotavirus disease, is caused by serotonin (5-hydroxytryptamine, 5-HT). 5-HT is secreted by enterochromaffin cells (EC) that can be directly infected with and replicate rotaviruses in humans. The 5-HT activates vagal afferent nerves connected to the nucleus of the solitary tract and area postrema in the brainstem structures associated with nausea and vomiting [65].

### 2.5. Immune Response to Rotavirus

The mechanisms responsible for immunity to rotavirus infections are not completely understood. Animal models have been useful in elucidating the role of antibodies and in exploring the relative importance of systemic and local immunity [81]. In humans, rotavirus infection has been shown to induce a good humoral immune response and protection increases with each new infection and reduces the severity of the diarrhea [82].

Primary rotavirus infections induce production of rotavirus-specific memory B and T cells [82]. Since the immunity against severe diarrhea in humans resulting from series of childhood rotavirus infections often wanes with age, elderly persons become more susceptible to rotavirus reinfection [83]. The significance of the systemic presence of IgA, IgG, and IgM antibodies towards protection against rotavirus infection in both humans and animals remains to be understood [81, 84]. However, it is known that maternal IgG antibodies may play a role in protecting infants under the age of three months from developing severe diarrhoea caused by rotavirus infections as evidenced by the neutralizing activity of antibodies detected from transitional milk and colostrum specimens [85]. Protection of neonates against rotavirus infection appears to be conferred by both transplacental acquired maternal antibodies and by antibodies and other factors in breast milk. Interestingly, rotavirus infection in neonates often results in asymptomatic infection unless novel serotypes emerge, and rotavirus can circulate silently in neonatal units [86].

### 2.6. Factors Affecting Disease Severity

The factors that influence the severity of the disease as well as pathogenesis are reduced intake of colostrum, age and health status of the calves, immune status of the dam, degree of exposure and virulence of virus, and the presence of secondary pathogens [87]. If rotavirus infection occurs in combination with *E. coli* or coronavirus, the mortality rate could be high. Several other factors like dehydration, unhygienic environment, temperature variations or chilling during winter, and high population density in farms may also enhance disease severity. However, the major stress factors that potentiate the infection have been found to be cold climate and marked fluctuations in the ambient temperature between day and night. An age-related resistance has also been observed. As there is competition between the rate of replication of rotavirus and replacement of enterocytes in older animals, highly virulent strains can only cause diarrhea in adult calves [77].

### 2.7. Clinical Features of Rotavirus Infection

#### 2.7.1. Symptoms in Animals

Rotavirus diarrhea in calves presents an acute disease having very short incubation period of 12–24 hours or at times ranging within 18–96 hours. Fortunately, most rotavirus infections are mild and self-limiting, although there is usually high morbidity. Variations in clinical disease observed in calves depend on a number of factors, including difference in virulence among rotavirus strains, age of the host, host immune status, dose of the inoculum, occurrence of mixed infections, environmental stress (weather conditions, housing, overcrowding), and nutrition, in addition to systemic consequences of electrolyte imbalances, fluid loss and metabolic acidemia, anorexia, profuse watery diarrhea, and various degrees of systemic dehydration. In severe cases, death occurs as a result of electrolyte imbalances, dehydration, and cardiac arrest [88].

### 2.8. Transmission

Rotaviruses are highly contagious, ubiquitous in the environment, and relatively resistant to disinfectants. The adult animals are the main source of infection in newborn animals, and serological surveys revealed that 50–100% of adult animals might show immune response against RVA. Young calves, especially aged 1–3 weeks, are most vulnerable to the rotavirus infection and infection rate declines as age of calf increases [89]. The infectious dose is low (as few as 10 particles) [90]; and the virus is shed in large quantities (as many as 10^11^ particles per gram of stool) both before the onset of symptoms and for several weeks afterward. The virus transmits through a fecal-oral route and calves are most often infected by contact with other calves, primarily or secondarily through objects, feed, and water. It has been proposed that calves can also be infected by virus shed by the dam at birth. The infected calves shed virus through the feces from the second day of infection and the shedding may last for 7-8 days. The virus primarily affects neonatal individuals, and calves more than 3 months of age are usually not affected. Rotavirus that infects calves causes often severe and sometimes life-threatening diarrhea [77].

Transmission to susceptible individuals occurs mainly by the fecal-oral route through direct contact with the rotavirus, including children and adults with asymptomatic illness and contact with contaminated fomites, food, water, and environmental surfaces [91, 92]. It has been reported that improvements in hand hygiene in hospitals can decrease the incidence in healthcare-associated rotavirus infections. It has also been suggested that aerosol transmission might be important. Evidence of the airborne spread of rotavirus gastroenteritis is primarily circumstantial, including the short incubation period (1–3 days) and the fact that the virus often presents in explosive outbreaks [78]. Rotavirus has also been detected in the respiratory secretions from a small number of patients, and cases of pneumonia have been described. Rotavirus epidemics exhibit a seasonal pattern [93]. In temperate climates, rotavirus infections peak in the winter months. Seasonality is less marked closer to the equator, but the disease is more common during drier and cooler months. Recent data suggest that the seasonality of rotavirus could have been changed by the introduction of rotavirus vaccines [94, 95].

### 2.9. Diagnosis of Rotavirus

Laboratory diagnosis of rotavirus is very important for management and control of outbreak of disease related with rotavirus infection in calves. Viral gastroenteritis is caused by different types of viral antigens like coronavirus, noroviruses, astroviruses, and adenoviruses. It is very difficult to diagnose specific causal agents by clinical examination, so laboratory diagnosis is vital for confirmatory diagnosis. This can be carried out by using various tests [5]. Rapid and accurate detection of the etiological agent is important to further contain the spread of infection in animals. Rotavirus is shed in high concentration in the stool (∼10^12^ viruses/gram) of children with gastroenteritis. Therefore, measurement of rotavirus antigen in the stool has been used to identify rotavirus infected patients. Generally, the diagnosis of rotavirus is based on isolation and identification of the virus in intestinal contents or feces [88]. Isolation of rotavirus has been performed in rotavirus-specific cell line MA-104 (Simian origin), and direct detection has been facilitated by electromicroscopy. Immunofluorescence test (IFT), immunoperoxidase test (IPT), and viral RNA-based PAGE have also been employed to detect the infectious agent. Latex agglutination test (LAT) has also been used for the rapid detection of rotavirus antigens [96, 97]. ELISA, being a highly sensitive and specific test, has been developed by many workers and used for the identification of rotaviruses [18].

#### 2.9.1. Antigen Capturing Enzyme-Linked Immunosorbent Assay (Ag-ELISA)

Ag-ELISA is an assay for rapidly detecting a pathogen in a clinical specimen based on antibody (e.g., monoclonal antibody) recognition of the target antigen [98]. It has antibody attached to a solid surface which can be a glass, plastic material, or membrane filter. This antibody captures the target antigen if present in the sample. Then, there will be a cascade of colorimetric reactions to verify capturing of the antigen and visualize the antigen-antibody reaction. Antigen can be quantitatively estimated as optical density (OD) measured by a spectrometry positively correlates with the amount of antigen. In some situations, the commercial kit may be expensive, particularly for veterinary medicine [5].

#### 2.9.2. Electron Microscopy (EM)

Electron microscopy (EM) is used for virus detection and identification based on morphological characteristics. There are two types of EM methods: direct EM and immune-electron microscopy (IEM) [99]. Two different staining techniques (positive and negative staining) are used to visualize the presence of target. In the direct EM, virus particles in a fluid sample matrix are applied directly to a solid support and then are visualized by EM after a contrast stain is applied. It is commonly referred to as “negative straining EM,” whereas positive staining is generally used in a thin-section EM on fixed tissues. In comparison, IEM has a higher sensitivity and specificity than direct EM as a specimen is incubated with antibody specific for the target virus in order to agglutinate the virus before staining. The visualization of viruses, particularly noncultivatable ones, is a major advantage of EM with rapid turnaround. Most of bovine enteric viruses, such as BRV, BToV, and BCV, are difficult to isolate or propagate in cell culture, but these viruses can be differentiated by their morphology under an electron microscope. The cost of electron microscopes and requirement of skilled laboratory personnel are still a challenge for the EM test being used as a routine diagnostic test [6].

#### 2.9.3. Isolation of Virus in Cell Culture

Virus isolation test is a confirmatory diagnostic test that is still considered as “gold standard” for detecting the presence of viral pathogens in specimens [6]. Cell culture techniques are commonly used for virus isolation for diagnostic purpose, as well as virus propagation for vaccine production or further virus characterization such as antigenic variation or gene sequencing [100]. The isolation of rotavirus in cell culture from fecal samples is the most conventional way of confirmatory diagnosis of rotavirus infection and gives the ultimate proof of virus association with the disease but it is less sensitive and is a laborious process. Isolation of BRV is performed in rotavirus-specific primary cell cultures (calf kidney cells) and cell lines (MA 104-Simian origin, MDBK, HT-29, and PK-15). Presence of virus is suspected by occurrence of cytopathic effect (CPE) including rounding and detachment of cells in cell culture system. Enhancement of CPE has been shown to be increased by incorporation of trypsin in the medium in minute quantities and by the pretreatment of fecal samples with trypsin [87]. The viability of target virus in a specimen is critical for the success of virus isolation [101]. Specimens should be kept at a low temperature and in a transport medium during shipping to a diagnostic laboratory and delivered to the lab as soon as possible after collection [101].

#### 2.9.4. Rotavirus dsRNA PAGE

The rotavirus dsRNA can be detected in clinical specimens by extraction of viral RNA and analysis by electrophoresis on a polyacrylamide gel followed by silver staining. During electrophoresis, the 11 segments of the rotavirus dsRNA, which are negatively charged molecules, separate according to size [102]. The patterns of dsRNA can be visualized in the gel by staining with silver nitrate, because silver ions form a stable complex with nucleic acids. The gel can be stored after staining. The migration patterns of the segments of rotavirus dsRNA allow the classification of rotavirus strains into the “short” and “long” electropherotypes [6].

#### 2.9.5. Polymerase Chain Reaction (PCR)

Polymerase chain reaction (PCR) is frequently used test method for detecting rotavirus. It is a thermocyclic enzymatic amplification of specific sequence of the target genes using a pair of oligonucleotide primers that hybridize on each cDNA strand of interest region in the genomic sequence. The detection of rotavirus dsRNA in fecal specimens consists of 4 steps: (i) viral dsRNA extraction, (ii) denaturation of the rotavirus dsRNA, (iii) reverse transcription of dsRNA, and (iv) amplification of cDNA by PCR; PCR consists of (a) heating the DNA to be amplified to separate the template strands, (b) annealing of two primers that are complimentary to the region to be amplified, (c) extension of the primers by a heat stable DNA polymerase enzyme that uses each DNA strand as template, and (d) repeating the process 30–40 times with the newly synthesized cDNA heat denatured and the enzymes extending the primers attached to the separated single DNA strand. After completion of the reaction, the PCR products can be visualized on an agarose or acrylamide gel by electrophoresis technique and special staining with ethidium bromide. Amplification of the target sequence is determined based on molecular size and/or sequencing of the PCR product [5].

#### 2.9.6. Rotavirus Genotyping Using RT-PCR

With regard to reverse transcription-polymerase chain reaction (RT-PCR), ever since the initial report by Kary Mullis and coworkers in 1986 about in vitro enzymatic amplification of specific DNA fragments from complex nucleic acid samples using PCR, a number of different applications of the technique have grown exponentially. A novel G-typing was first reported by using method based on RT-PCR amplification of the VP7 gene with type-specific primers [103]. In addition to this, some study used RT-PCR for serotyping of rotavirus virus and reported that six VP7 serotypes or G types (G1-G4, G8, and G9) occur in group “A” human rotaviruses [104]. In their study, they could type about 89% of the samples [104]. Another report compared the tests and used PCR for identifying serotypes of human and bovine rotaviruses, and PCR was shown to be more sensitive (93%) than ELISA (82%) [105].

RT-PCR is more sensitive (100%) and specific (99%) in comparison to ELISA and PAGE. As against RNA electrophoresis and ELISA, it provides a more accurate detection of rotaviruses by 18.8% and 26.5%, respectively. In recent reports, it has been shown that increased detection and quantification of group “A” rotavirus can be done by real-time RT-PCR. For easy screening of the fecal samples for rotavirus A, a diagnostic RT-PCR assay was developed by targeting the group specific VP6 gene [106].

One researcher developed a one-step multiplex RT-PCR method for the simultaneous detection of five viruses causing diarrhoea in adult cattle, i.e., bovine group A rotavirus (rotavirus A), bovine group B rotavirus (rotavirus B), bovine group C rotavirus (rotavirus C/GCR), bovine coronavirus (BCV), and bovine torovirus (BToV) [107]. In this report, the one-step multiplex RT-PCR was found to have higher sensitivity to detect rotavirus A than a single RT-PCR with conventional primers. The results indicate that the one-step multiplex RT-PCR developed can be used for the detection of rotavirus A, rotavirus B, rotavirus C, BCV, and BToV and can be expected to be a useful tool for the rapid and cost-effective diagnosis and surveillance of viral diarrhea in adult cattle [94].

#### 2.9.7. Real-Time PCR

Real-time PCR is a PCR method which amplifies the target sequence and also quantifies the amount of the target with higher sensitivity. Real-time reverse transcription-PCR is a high-throughput robust easy-to-perform, quantitative, sensitive, and specific assay to detect viral nucleic acids [108]. Multiplex real-time PCR based on SYBR Green and TaqMan assay have been developed for detection of group A human rotavirus. Multiplex real-time PCR has also been described to detect rotavirus along with other enteric pathogens in bovine fecal samples [109]. Compared to conventional RT-PCR, real-time RT-PCR has been shown to be more rapid and more sensitive for the detection and quantitation of rotavirus [106, 110]. For rapid diagnosis of rotavirus in fecal samples, a SYBR Green based real-time PCR assay was developed targeting the NSP4 gene [106].

One of the primary advantages of real-time PCR is the ability to identify amplified fragments during the PCR process. Real-time PCR measures the amount of the product. Standard PCR requires post-PCR analysis, possibly agarose gel electrophoresis. The use of probe hybridization is often used for characterization of the product by its sequence. Though this method is more reliable and informative, it is time-consuming and expensive. ELISA detections are also time-consuming. Real-time PCR eliminates these needs. Amplicon recognition is achieved by monitoring the accumulation of specific products during each cycle. Another advantage of real-time PCR over standard PCR is that the entire process from amplification to analysis is performed in the same tube. This differs from standard PCR where the PCR product is moved and manipulated into other formats. As a result, there is a decreased possibility of contaminating the product with real-time PCR methods [106, 110].

#### 2.9.8. Restriction Fragment Length Polymorphism (RFLP)

Restriction Endonuclease (RE) analysis of field rotaviruses is a powerful tool to understand genomic diversity of rotaviruses circulating in environment. Apart from proving useful in monitoring the extent of genetic variation among rotavirus strains within a population, RFLP may also prove valuable in the examination of interspecies transmission and possible source of origin of rotavirus strain. Chang et al. [111] used RFLF for P and G genotyping of bovine rotavirus A. Gouvea et al. [103] analyzed 194 strains of rotavirus A representing all known G types digestion with three restriction enzymes (Sau96I, BstYI, and HaeIII) by direct digestion of amplified cDNA copies or by deduction of the restriction patterns from known sequences. Digestion with Sau96I and HaeIII identified restriction sites commonly used for all, or mostly for all, strains of rotavirus studied, whereas BstYl was the most discriminating among rotavirus strains.

#### 2.9.9. Reverse Transcription Loop-Mediated Isothermal Amplification (RT-LAMP)

Nemoto et al. [112] developed RT-LAMP for detection of equine rotavirus targeting P [12], the most predominant P genotype worldwide. The results indicated that the RT-LAMP assay was specific for equine rotavirus and was found more sensitive than semi-nested RT-PCR. Because RT-LAMP is easy to perform without the need for a thermal cycler or gel electrophoresis, the RT-LAMP assay should be applicable to diagnosis of equine rotavirus infections in diagnostic laboratories.

#### 2.9.10. Hybridization Assays

The assessment of the genetic variability of rotavirus by hybridization assay, including blot techniques such as northern and southern blot and also liquid assays, has been an alternative approach to PCR assays. Most northern blot and liquid hybridization assays have utilized cDNA or ssRNA probes synthesized from all segments in a single hybridization reaction and thus limit the amount of segment specific information available from the test [113]. Nonradiolabeled cDNA probes have been used for G and P genotyping of bovine rotavirus A [114].

#### 2.9.11. Latex Agglutination Test (LAT)

LAT is in principle similar to ELISA test [115]. Antigen or antibody is coated on the surface of latex particles, which captures antibody and the target antigen, respectively. The test has been applied for the detection of a wide range of targets, such as bacteria, virus, hormones, drugs, and serum protein [116]. Latex particles are made of synthetic rubber and emulsified as billions of micelles of the same size of a desired diameter. Usually, the size of particles ranges between 0.05 and 2 *μ*m in diameter, and the presence of sulfate ions provides an inherent negative surface charge to the particles [117]. This prepared latex particle can be further functionalized by special processing, such as amidation, amination, carboxylation, hydroxylation, or magnetization, to increase their binding stability and analytic attachment depending on the purpose of test [117]. The latex agglutination test is frequently employed in diagnostic lab, because it can be a semi-quantified test and is relatively cheap with rapid turnaround. Caution should be taken in interpreting marginal results as false positive/negative results frequently occur due to nonspecific binding or interference [115].

### 2.10. Treatment

There is no specific treatment for rotaviral infections. Treatment is based in providing supportive care and managing clinical signs and potential complications. In livestock and companion animals, fluid administration is essential to replace losses from diarrhoea or vomiting, to correct acidosis and to restore electrolytes imbalance. Adequate sodium concentration and appropriate glucose to sodium ratios are the most important components of an efficient rehydration solution [118]. In young animals, administration of fluids can be performed by means of oesophageal catheter; in older animals, intravenous administration is preferable. In affected piglets, administration of a plasma protein mixture, consisting of immunoglobulins, growth factors, and other biologically active peptides, has been advocated to enhance small intestine recovery [119].

### 2.11. The Zoonotic Potential of Rotavirus

Rotaviruses have a wide host range, infecting many animal species as well as humans. As it was found that certain animal rotavirus strains had antigenic similarities to some human strains, speculation increased about whether animals play a role as a source of rotavirus infection in humans. There is however an alternative view that animal rotaviruses can indeed infect humans and cause disease whenever the chance exists. This is based on the identification of unusual rotavirus types, with properties of strains more commonly found in animals, which were isolated from various cases of human infection. These unusual human rotavirus types may have arisen either as whole virions or as genetic reassortants between human and animal strains during coinfection of a single cell [120]. The segmented nature of the genome suggests that, like other viruses with segmented genomes such as influenza virus, rotaviruses are able to form new strains by a mechanism of reassortment. Reassortment can occur when two rotaviruses of two different strains infect the same cell, and during replication and packaging they exchange genome segments [121]. The 11 genome segments of the parental virus strains can theoretically reassort into 2048 [121, 122] different possible genome constellations, if reassortment is random.

Gouvea and Brantly [123] hypothesized that rotaviruses exist as mixed populations of reassortants and that reassortment was the driving force behind diversity. A prerequisite of diversity is cocirculation of many different rotavirus types in a population; and more diversity, as well as more frequency of uncommon strains, is seen in years with the highest number of cocirculating strains [124]. Gouvea and Brandtly considered that mixed populations of rotaviruses are being continually propagated in human and animal hosts, resulting in new and diverse progeny populations of rotavirus. With regard to new rotavirus strains arising through reassortment, a concept of zoonotic genes may be developed. These can be defined as genes originating in animal rotaviruses which can interact with genes of human rotaviruses, to form infectious rotavirus particles which are serially propagated in the human population [3].

Until recently, specific rotavirus types have been associated with specific animal species. For example, human rotaviruses most commonly belong to G types 1–4 and P types [4] and [8] [125], whereas bovine rotaviruses most commonly belong to G types 6, 8, and 10 and P types [1], [5], or [11] [126]. The rotaviruses have been characterized, and the host species specificity of P and G types has become less distinct. Human group A rotavirus strains that possess genes commonly found in animal rotaviruses have been isolated from infected children in both developed and developing countries. Strains such as G3 (found commonly in species such as cats, dogs, monkeys, pigs, mice, rabbits, and horses), G5 (pigs and horses), G6 and G8 (cattle), G9 (pigs and lambs), and G10 (cattle) have been isolated from the human population throughout the world [127].

Groups A to C have been shown to infect both humans and animals [128]. Members of Rotavirus Group A are classified according to their glycoprotein (G) structures, namely, G (G1, G2, G3,…, Gn) genotypes, and their protein cleavage (P), namely, P (P [1], P [2], P [3],…, P [n]) genotypes [129]. Currently, 36 G genotypes and 51 P genotypes have been identified in humans and animals worldwide [130]. G and P type combinations which are found in man have also been found in animal species. For example, G10P [11] was found in American and Canadian cattle by Lucchelli et al. [131]. and in Indian cows and buffaloes by Gulati et al. [132] G3P[6] and G4P[6] were found in pigs in Poland and the USA and G1P [8] and G5P [8] were found in pigs in Brazil by Santos et al. [133]. The emerging G9 strains 26–28 may have arisen in humans through transfer from animals. They have been found in lambs and pigs [133, 134].

Epidemiologically, there exists evidence for zoonotic transmission of rotaviruses. Human Group A rotavirus strains possessing genes commonly found in animal rotaviruses have been isolated from infected children in both developed and developing countries. Strains such as G3 (found commonly in species such as cats, dogs, monkeys, pigs, mice, rabbits and horse), G5 (pigs and horses), G6 and G8 (cattle), G9 (pigs and lambs), and G10 (cattle) have been isolated from the human population through the world [127].

In humans, they appear to cause more severe symptoms than the common rotavirus strains [135], which might be due to less immunity to these emerging strains, or to greater virulence being conferred by their genetic makeup. Several studies have indicated symptomatic infection of humans by animal viruses. Nakagomi and Nakagomi, [136] reported that almost all gene segments of the rotavirus G3 strain (AU228) isolated from a child with a pet cat were identical to those of a feline rotavirus strain (FRV-1). Strains very similar to this may have become established in humans [137]. A three-week-old baby in an Israeli household which had a young dog (<6 months old) was infected with an animal rotavirus G3 strain [113]. Das et al. [138] reported that a G8 rotavirus which had widely circulated in newborn infants in India, causing asymptomatic infection, had VP7 and VP4 gene sequences which were identical to those of a bovine rotavirus strain.

Some feline and canine rotavirus strains have spread into human populations as whole virions, bovine rotaviruses were involved in reassortment with human rotaviruses, leading to the emergence of unusual strains in various parts of the world. Apparent dual infection with human and animal rotaviruses has been observed recovered G1P [5] and G1P [8] strains from an infant with severe diarrhoea. The G1P [5] rotavirus was genotypically similar to bovine strains. It was not isolated from the infant in high titer and possibly had little, if any, effect on the child's disease. Nonetheless, it would have had the potential to reassort with the coinfecting strain [136].

### 2.12. Control and Prevention of Rotavirus Infections

Rotaviruses are infectious and comparatively resistant to inactivation by chemical disinfectants and antiseptics. Control and prevention measures against rotavirus infection are not so easy for its mass distribution and tendency to stability in different climate situation and are shed in high concentrations in feces of infected animals. The primary strategy to reduce the burden of rotavirus infections is vaccination. Vaccination protocol differs from the approaches implemented to protect infants and children against rotavirus disease [79].

In humans, the primary objective is the reduction of maternal antibody level by the age of 4–6 months; active immunity induced by vaccination is elicited to last during the first few years of children lives when the risk of severe infections is the greatest. In order to decrease the incidence of disease in the herd, a good producer should maximize colostrum transfer, increase environmental sanitation, reduce stressors such as overcrowding or poor nutrition, and vaccinate bred cows for rotavirus at 60 and 30 days before calving [62].

First-milking colostrum is a source of nutrients and of passively absorbed maternal antibodies, critical to protect the newborn calf against infectious disease in the first weeks and months of life. The calf is born without most antibodies, including those that fight the infectious agents which cause diarrhea. The calf will acquire these antibodies only from colostrum [139]. Because of this, any effort to prevent diarrhea by vaccinating cows is wasted unless the calf actually receives colostrum, preferably before it is two to four hours old. As the calf grows older, it rapidly loses its ability to absorb colostral antibodies. Colostrum given to calves that are more than 24 to 36 hours old are practically useless; antibodies are seldom absorbed this late in life. The neonatal calf should ideally receive 2 to 3L (for beef calves) or 3 to 4L (in dairy calves) of colostrum within the first 6 hours after birth. The colostrum contains antibodies, immune cells (neutrophils, macrophages, and T and B cells), complements, lactoferrin, insulin-like growth factor-1, transforming growth factor, interferon, and nutrients [140].

To improve the passive immunization of calves against rotavirus and coronavirus as well as against different strains of *E. coli* vaccination of the pregnant dam can be proposed. Usually cows are vaccinated twice (6 to 8 and 2 to 3 weeks) before parturition to stimulate the production of specific antibodies. The primary function of colostrum is to enhance the calf's immune system through the passive transfer of both antibody and cell-mediated immunity. Ideally, calves should receive colostrum from their dams although colostrum from several cows is often mixed and administration of colostrum feeding results in the transmission of BVDV, bovine leukemia virus, and John's disease that can be spread by infected or purchased colostrum [141].

Specific IgG present in colostrum may protect against the more common enteropathogens causing calf diarrhea, such as rotavirus, coronavirus, and *E. coli*. Although vaccination of the dam prior to calving may boost colostrum IgG concentrations [118, 142], vaccinate the cows and pregnant heifers with any necessary calf diarrhea vaccines well prior to calving. Vaccines that contain rotavirus, coronavirus, and the K99 *E. coli* antigens can be helpful in preventing calf diarrhea. These are best given to the cow prior to calving so it can make antibodies and secrete them into the colostrum. When the calf ingests this enriched colostrum, it will be protected against these major agents [143]. In animals, the concept of passive immunization is based on maternal antibodies that are transferable through the placenta or are secreted in the colostrum providing transient protective immunity to offspring against clinically manifest RVA infection. Rotavirus vaccines have been developed to control the neonatal calf diarrhea associated with rotavirus infection. Most of the commercial vaccines are combined with more than one agent [144].

Commercial RVA vaccines are administered parenterally to cows and sows during the late stage of gestation, in order to elicit a strong maternal immunity that is readily conferred to newborn animals. Some studies have demonstrated vaccine failure or breakthroughs that have been related to a number of factors, including inadequate managing conditions of animals or antigenic differences between vaccine and field RVA strains, even if vaccine and field strains shared partially their surface antigen specificities. Moreover, optimum management and hygienic practices can minimize the incidence of rotaviral diarrhea in farm animals. To control secondary bacterial infection, antibiotics and fluid and electrolyte therapy to restore the fluid reserve have to be given due importance so that the mortality rate in calves could be minimized [87].

## 3. Conclusion and Recommendations

Diarrheal disease caused by rotavirus poses a great health problem in calves that interrupts production benefits with reduced weight gain and increased mortality, and its potential for zoonotic spread [68]. Rotavirus is a major pathogen responsible for diarrheal disease in calves resulting in loss of productivity and economy of farmers. However, various facets of diarrheal disease caused by rotavirus in calves in world are inadequately understood. Awareness of the advantage of colostrum feeding is not enough, but also times of colostrum administration to neonate calves are crucial for the ultimate development of immune status against pathogens including rotavirus infection. Calving areas should have well-drained grass lots or pastures visible from the barn area and calving areas should be selected or landscaped to allow for adequate drainage. Enteric disease like rotavirus infection is a vital health problem in calves that interrupts production benefits with reduced weight gain and increased mortality, and the virus potential for its zoonotic spread; it is imperative to determine the disease burden and responsible risk factors. This is very useful to execute effective preventive measures such as practicing early colostrum feeding in newborn calves, vaccination in dams, and improving livestock management. Rearing healthy dairy calves to weaning time requires maximizing the calf's level of immunity against disease while minimizing its exposure to infectious agents. Based on the above conclusion, the following recommendations were forwarded:

- Awareness creation for researcher and government regarding the effect of rotavirus infection in calf's health and growth performance and national economy is very important
- Further studies of rotavirus infection in calves covering larger areas of the country need to be conducted

## Figures and Tables

**Table 1:** Rotavirus proteins, genome segments, and structural localization.

Protein	dsRNA segment no	Location in virus capsid	Function	Numbers of molecules/virion
VP1	1	Core	dsRNA synthesis (RNA-dependent RNA polymerase)	12
VP2	2	Core	Inner shell protein	120
VP3	3	Core	Capping enzyme	12
VP4 (cleaved to VP5 and VP8)	4	Outer capsid	Viral attachment, P-type neutralization antigen	120
VP6	6	Inner capsid	Middle shell protein	780
VP7	9	Outer capsid	G type neutralization antigen	780
NSP1	5		INF antagonist	—
NSP2	8		Viroplasm formation	—
NSP3	7		Enhance viral mRNA synthesis, associated with systemic spread	—
NSP4	10		Outer capsid assembly, regulate calcium homeostasis, enterotoxin	—
NSP5	11		Viroplasm formation	—
NSP6	11		Viroplasm formation	—

Source: [22].

**Table 2:** Rotavirus group detected so far in different mammalian and/or avian host species.

Rotavirus group/species	Host species
A	A wide variety of mammalian and avian species
B	Humans, cattle, goats, pigs, rats, and sheep
C	Humans, cattle, dogs, goats, juvenile ferrets, and pigs
D	Chicken and turkey
E	Pigs
F	Chicken
G	Chicken
H	Humans and pigs

Source: [34].

**Table 3:** Common RVA G and P genotypes found in humans and animals.

Host species	Typical RVA VP7 and VP4 genotypes
Cattle	G6, G8, G10, P [1], P [5], P [11]
Pigs	G3-G5, G9, G11, P [6], P [7]
Horses	G3, G14, P [12]
Cats and dogs	G3, P [3], P [9]
Humans	G1-G4, G9, G12, P [4], P [6], P [8]

Source: [34].

**Table 4:** Geographic distribution of rotavirus serotypes.

Region	Rotavirus serotypes					
	G1P [8] (%)	G2P [4] (%)	G3P [8] (%)	G4P [8] (%)	G9 (%)	Other (%)
North America	73	11	6	1	3	5
South America	34	23	2	9	16	11
Europe	72	9	2	11	4	1.4
Australia	82	14	1	2	0.5	0.1
Asia	34	13	1	20	12	14
Africa	23	2	21	4	7	27
Taiwan	40	80	27	0	18	8

Sources: [39, 40].

**Table 5:** Prevalence of rotavirus infection in animals.

Country	Prevalence rotavirus (%)	Reference
Western Algeria	14.63	Ammar et al. [53]
Northern India	26.8	Jindal et al. [54]
Ethiopia	16.7	Abraham et al. [15]
India	15.68	Rai et al. [55]
Iraq	15.5	Al-Robaiee & Al-Farwachi [56]
Brazil	20.2	Alfieri et al. [1]
Tunisia	22.8	Zrelli et al. [57]
Brazil	25.1	Langoni et al. [58]
Algeria	21.84	Kam et al. [59]
England	42	Reynolds et al. [12]
Scotland	50	Snodgrass et al. [60]
Spain	42.7	De la Fuente et al. [61]
Australia	79.9	Izzo et al. [62]

## Data Availability

The datasets used and/or analyzed during the current study are available from the corresponding author on reasonable request.

## Abbreviations

Ag: Antigen
Ag-ELISA: Antigen capturing enzyme-linked immunosorbent assay
BCoV: Bovine coronavirus
BRoV: Bovine rotavirus
cDNA: Complementary DNA
CPE: Cytopathic effect
DMEM: Dulbecco's modified eagle medium
DNA: Dioxy nucleotide ribonucleic acid
DNTPs: Dioxy nucleotide triphosphates
dsRNA: Double stranded RNA
ELISA: Enzyme-linked immunosorbent assay
MDBK: Madin-Darby bovine kidney cell
mRNA: Messenger RNA
NSP: Nonstructural proteins
PBS: Phosphate-buffered saline
PCR: Polymerase chain reaction
pH: Power of hydrogen
RNA: Ribonucleic acid
rpm: Revolution per minute
RT-PCR: Reverse transcriptase polymerase chain reaction
UK: United Kingdom
VP: Structural proteins.
